# Hydrogen peroxide as a mitigation against *Microcystis* sp. bloom

**DOI:** 10.1016/j.aquaculture.2023.739932

**Published:** 2023-12-15

**Authors:** Pok Him Ng, Tzu Hsuan Cheng, Ka Yan Man, Liqing Huang, Ka Po Cheng, Kwok Zu Lim, Chi Ho Chan, Maximilian Ho Yat Kam, Ju Zhang, Ana Rita Pinheiro Marques, Sophie St-Hilaire

**Affiliations:** Department of Infectious Diseases and Public Health, Jockey Club College of Veterinary Medicine and Life Sciences, City University of Hong Kong, Hong Kong, China

**Keywords:** Aquaculture, Harmful algal bloom, Pond water, Hydrogen peroxide

## Abstract

*Microcystis* sp. is a harmful cyanobacterial species commonly seen in earthen ponds. The overgrowth of these algae can lead to fluctuations in water parameters, including DO and pH. Also, the microcystins produced by these algae are toxic to aquatic animals. This study applied hydrogen peroxide (7 mg/L) to treat *Microcystis* sp. in a laboratory setting and in three earthen pond trials. In the lab we observed a 64.7% decline in *Microcystis* sp. And in our earthen pond field experiments we measured, on average, 43% reductions in *Microcystis* sp. cell counts within one hour. The treatment was found to eliminate specifically *Microcystis* sp. and did not reduce the cell count of the other algae species in the pond. A shift of the algae community towards the beneficial algae was also found post-treatment. Lastly, during the pond trials, the gill status of Tilapia and Giant tiger prawn were not affected by the H_2_O_2_ treatment suggesting this may be a good mitigation strategy for reducing cyanobacteria in pond aquaculture.

## Highlights

• *Microcystis* sp. blooms are commonly found in earthen aquaculture ponds and can affect fish health and public food safety.
• Hydrogen peroxide successfully controlled the cyanobacteria in ponds without affecting beneficial algae and aquatic animals.
• Pros and cons of the hydrogen peroxide treatment against harmful algal bloom and how to apply this mitigation.

## 1 Introduction

Harmful algal blooms (HAB) cause millions in losses annually (Anderson, 2009; Heil and Muni-Morgan, 2021). In aquaculture, HABs often lead to massive fish death due to the depletion of oxygen (Belfiore et al., 2021), poisoning by toxins (Anderson, 2009), or respiratory dysfunction by obstruction of the gills (Díaz et al., 2019).

Certain environmental characteristics such as warm water temperatures, high light intensity, prolonged light photoperiods, and eutrophication, favour the growth and dominance of algae species, which can result in the formation of HABs (Al Shehhi et al., 2014; Pal et al., 2020). Specific types of algae that fall within the HAB classification include Dinoflagellates, Diatoms, Haptophytes, Raphidophyceans, Cyanobacteria, or Macroalgae (Granéli and Turner, 2006). *Microcystis* sp. is a frequently found cyanobacterium dominating fresh water bodies during the algal bloom season (Ruangrit et al., 2013). Blooms with this algae can create fluctuations in the water quality in ponds, especially in DO and pH (Belfiore et al., 2021; Díaz et al., 2019; Liu et al., 2020), which could affect the growth of aquatic animals or cause mortalities (Masango et al., 2010; Mohan et al., 2020). *Microcystis* sp. blooms can hinder light penetration, compete for nutrients, and inhibit the growth of other vegetation in earthen ponds. The toxin, microcystin produced by these algal species is harmful to aquatic organisms and could accumulate in the food web (Braga et al., 2018; Ruangrit et al., 2013) leading damage to the consumer's liver (Díaz et al., 2019; Massey et al., 2018).

Harmful algal blooms mitigations can be achieved by physical, biological, and chemical measures (Balaji-Prasath et al., 2022). Physical methods of reducing algae include flocculation, dispersing clay, shading, and ultrasonication (Li and Pan, 2013; Park et al., 2017; Yu et al., 2017). Those methods usually inhibit the photosynthesis of the algae by either blocking the light directly or disturbing the buoyancy of the algae, leading to sedimentation. Physical mitigation strategies generally have a low impact on the ecosystem and environment. However, the equipment needed to carry out these practices are often expensive, and the machines may not work in certain ponds (i.e., may require a certain depth). Biological mitigation methods include strategies that involve consumption of algae. For example introducing zooplankton (i.e., cyclopoid, copepod, calanoid, *Daphnia* sp.), fish (i.e., Grass carp, bighead carp, silver carp, Tilapia), and mussels (Trapp et al., 2021). Fungi, Bacteria, and Viruses can also be used to lyse the cyanobacteria or degrade the toxins produced by cyanobacteria (Pal et al., 2020). Biological methods are generally low in cost, but often less effective than chemical methods. Chemicals to treat algae include, but are not limited to copper sulphate, chelated copper complexes, diquat (for filamentous algae), flumioxazin (for filamentous algae), Sodium carbonate peroxy-hydrate (work best on Filamentous blue-green algae), Mono(*N*,*N*-dimethylalkylamine) salt (for Planktonic/ macroalgae), triosyn, and hydrogen peroxide (Pal et al., 2020). They are overall very effective but usually only kill algae through direct contact (Kang et al., 2022), which can be problematic as it requires good dispersion of the products during their administration. Further, some chemicals, such as copper sulphate can accumulate in the environment and cause pollution problems (Tunçsoy and Erdem, 2018).

Previous studies on the effect of hydrogen peroxide (H_2_O_2_) against *Microcystis* sp. showed a low effective concentration range: of 4–20 mg/L (Liu et al., 2017; Mikula et al., 2012; Yang et al., 2018). The major advantages of using H_2_O_2_ to reduce cyanobacteria blooms include its rapid degradation into water and oxygen (Arvin and Pedersen, 2015), and it is easy to obtain. Further it may be selective for killing *Microcystis* sp.. Many of the studies published on the efficacy of H_2_O_2_ were conducted in a laboratory (Buley et al., 2023; Chen et al., 2021; Guo et al., 2021; Mukai et al., 2019), so the effect might differ in field applications.

In this study, we assessed the use of H_2_O_2_ to remove *Microcystis* sp.. We hypothesized that a 7 mg/L H_2_O_2_ treatment could eliminate *Microcystis* sp. without harming other algae species and aquatic animals.

## 2 Materials and methods

### 2.1 Lab-scale experiment

Twenty litres of pond water were collected from Au Tau Agriculture, Fisheries and Conservation Department (AFCD), Hong Kong SAR, China. The pond water was stored at 4 °C overnight for layer separation. *Microcystis* sp. were isolated from the surface layer and *Scenedesmus* sp. were collected from the middle layer of the pond water. Both layers of pond water were then separately passed through a 120 μm filter followed by a second filtration step through a 37 μm mesh which collected the algae. The algae stocks were then incubated at approximately 26 °C on Blue Green medium number 11 (BG-11) culture plates as described (Duong et al., 2012; Yang et al., 2018). Single colonies of *Microcystis* sp. and *Scenedesmus* sp. as identified under the microscope using morphological characteristics were sub- cultured on BG-11 for use in the following experiment. Both algae stocks were amplified in 100 mL conical flasks using BG-11 media at 26 °C.

*Microcystis* sp. and *Scenedesmus* sp. were added to twelve 500 mL bottles of BG-11 media to mimic their concentration ratio in the original pond. The twelve culturing bottles were divided into either control or treatment groups. Both groups were incubated with an 11 h:13 h light-dark cycle which used two 18 W red (450-470 nm) and blue (620-660 nm) combined LED light strips with a ratio of 5:1 (red-to-blue) and with 361 μmol/m^2^/s photosynthetic photon flux density (PPFD). PPFD was measured using a PAR Meter (APM092, KUNSHAN AST Optoelectronics CO., LTD, China). The photoperiod setting was according to the photoperiod during the algal bloom season in Hong Kong.

The treatment group was treated with 7 mg/L of H_2_O_2_ at time 0. Water parameters including dissolved oxygen, temperature, pH, nitrate-N, total ammonia and viable chlorophyll-a were measured at six time-points during the experimental period of fourteen days (Before the treatment: Day 0; after the treatment: 1 h, 1 day, 3 days, 7 days and 14 days) in both the treatment and the control groups. H_2_O_2_ was measured using hydrogen peroxide test paper (High Accuracy Hydrogen Peroxide Test Strip, Guangdong Huankai Microbial Sci. & Tech. Co.,Ltd., China) before the treatment: Day 0; and one hour, one day and three days after the treatment. Dissolved oxygen and temperature were measured with a handheld optical dissolved oxygen meter (model ProSolo with ODO probe, YSI, USA); pH and nitrate-N were measured with a portable parallel analyser using the pH probe and Nitrate probe respectively (SL1000 – PPA, Hach, USA); total ammonia was tested with a portable parallel analyser using total ammonia Chemkey® 120 reagents (SL1000 – PPA, Hach, USA); and viable chlorophyll-a concentration was assessed with a portable chlorophyll fluorometer (Model: ET1301, Shanghai 115 Euro Tech Ltd., China). During the six time-points, 10 mL of water sample were collected from each bottle. The samples were centrifuged at 3200 ×*g* for 30 min, re-suspended with PBS to make 100× concentrated samples and stained with Lugol's iodine (1% final concentration) (Olsen et al., 2016). Algae cell number were counted in 4 sets of 16 squares on a hemocytometer under a light microscope (400×). The algae counts were categorised as *Microcystis* sp., or *Scenedesmus* sp..

Counts of *Microcystis* sp. and *Scenedesmus* sp., viable chlorophyll-a, and water quality parameters were compared between control and treatment flasks at each time point. A Wilcoxon rank-sum test was used at each time point to determine whether the differences between outcomes in the treatment and control groups were statistically significant. Differences were considered significant if the *P*-value <0.05. We used a non-parametric statistical analysis because the data did not meet the assumptions of the ANOVA test. All statistical analyses were conducted in R (Team, 2022).

### 2.2 Pond studies

We applied H_2_O_2_ to ponds at an experimental site in Au Tau, Yuen Long District, Hong Kong, which had algal blooms with a cyanobacteria species *Microcystis* sp.. A total of 3 pond studies were conducted. The ponds were about 30 m × 20 m and 1.5 m deep. There were no aquatic animals in the first pond; while there were approximately four hundred jade perch in the second pond, and around five thousand giant tiger prawns in the third pond.

**Fig. 1:**
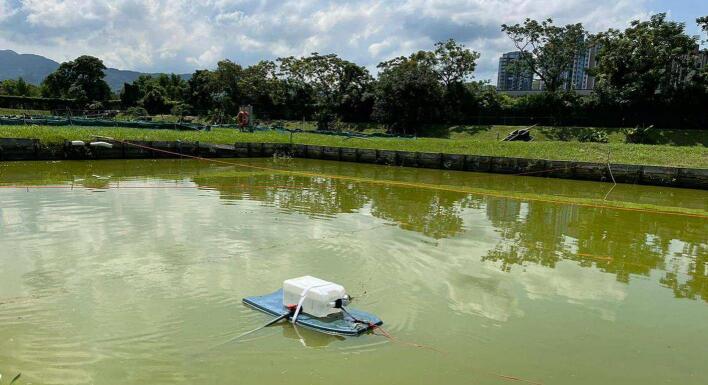
Floating hydrogen peroxide dosing device used for the even distribution of the H_2_O_2_ treatment to the targeted pond.

Hydrogen peroxide treatments at a concentration of approximately 7 mg/L (around 19 L of 30% Hydrogen Peroxide) were applied to all three ponds after the pre-treatment assessments. A second dose of H_2_O_2_ was applied on day 7 in the second trial. The latter was done to further reduce the *Microcystis* sp. regrowth rate. The treatments were applied to the ponds using a floating 20 L hydrogen peroxide dosing device, which had perforated pipes to allow even distribution of the chemical (Fig. 1).

In the first pond, two water samples were collected from the two diagonal corners and from the centre of the ponds (*n* = 3), and at all six time-points, similar to what was described in the laboratory experiment (before the treatment: Day 0; After the treatment: 1 h, 1 day, 3 days, 7 days and 14 days). Water quality parameters were measured, including H_2_O_2_ concentration, dissolved oxygen, temperature, pH, nitrate-N, total ammonia and viable chlorophyll-a. Additionally, 100 mL of water sample were collected from each sampling site at each time-point and transported to the laboratory at City University of Hong Kong for the algal content measurement. The samples were then concentrated, stained and counted following with the same protocol as described in the laboratory study. The two samples were averaged to provide a mean estimate at each sample location. Water quality parameters were described using boxplots.

In the second pond, two water samples were collected from all four corners and from the centre of the pond (*n* = 5), at 9 time-points (i.e. before the 1st treatment on Day −7, Day −5, Day 0; After the 1st treatment at 1 h, 1 day, 3 days, and 7 days; after the 2nd treatment at Day 7 + 1 h and 14 days). Water quality parameters and algae content were measured in a similar manner as described in the first pond trial. We also measured phosphate concentrations. Furthermore, in this pond five fish were netted and examined for external lesions and gill health on Day 0, 1 h after the treatment, Day 1 and Day 14. Gills were sectioned for histological examination using hematoxylin-eosin stain. One gill section from each of the five sampled fish were examined under the light microscope (20×). The number of dead fish during the experimental period were also recorded.

In the third pond, two water samples were collected from all four corners and the centre of the pond (n = 5) at 8 time-points (i.e., before the 1st treatment: Day −7, Day −5, Day 0; After the 1st treatment: 1 h, 1 day, 3 days, 7 days and 14 days). Water quality parameters and algae content were measured as described above. In addition, we added a measure of water alkalinity to this trial. Five giant tiger prawns were netted for gills health examination on Day 0, 1 h after the treatment, Day 1 and Day 14 using the same method as described for fish.

Separate Bayesian change point analyses were conducted for the *Microcystis* sp. counts over time to detect changes based on posterior probabilities (Erdman and Emerson, 2008). For this analysis we limited our data to the first 7 days post treatment because we only had one pond where we performed a second treatment (2nd pond trial). Analyses were performed in the R statistical programming language using the *bcp* package (Erdman and Emerson, 2008; Team, 2014). We repeated the Bayesian change point analyses on the count data for all other algae.

To determine if there was a significant treatment effect on the *Microcystis* sp. counts within the first three days after applying H_2_O_2_ we conducted a metanalysis using the results of time-series generalized log-linear models conducted on the three separate trails. Time-series GLMs were built using the *tsglm* and the *tscount* (Liboschik et al., 2017) in R software version 1.4.3, (Team, 2022). These analyses estimated the β-coefficient of the treatment effect over the defined time period (i.e., 1 h after the treatment, day 1 and day 3). We assumed the response variable for *Microcystis* sp. counts followed a Negative Binomial distribution to allow overdispersion. For our Metanalysis, we averaged (weighted by the standard error) the treatment effect coefficient from the three models to provide an overall estimate of the effect using the *rma* function of the package *metafor* (Wolfgang, 2010) in R.

## 3 Results

### 3.1 Laboratory experiment

**Fig. 2:**
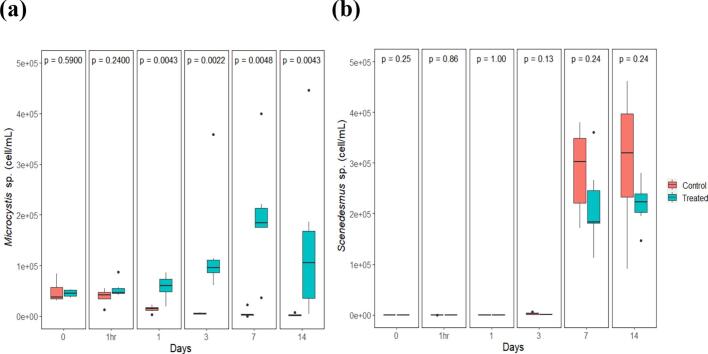
(a) *Microcystis* sp. cell counts over time in the control and treatment groups presented in box plots with median and 25–75 percentiles. The lines represent all the data range except extreme values, which are depicted with dots. *P*-value associated with the Wilcoxon rank-sum test indicated at the top of the panel. (b) Box plots of *Scenedesmus* sp. cell counts over time in the control and treatment groups with *P*-value associated with the Wilcoxon rank- sum test indicated at the top of the panel.

In the laboratory experiment, treatment and control groups had a similar initial *Microcystis* sp. count (47,867 ± 8599 and 45,104 ± 2947 cell/mL respectively) (Fig. 2A). For treated samples, there was a visible reduction in counts of *Microcystis* sp. over time, the first and most pronounced of which happened at 1 h and day 1, with a decrease of 64.7% in mean counts, followed by a decrease of 62% between days 1 and 3. In contrast, *Microcystis* sp. counts increased steadily over time in the control groups, with the largest spike (i.e. 135%) in *Microcystis* sp. counts occurring between day 1 and 3. This translated into a change in mean values of 57,692 ± 9742 cell/mL to 135,029 ± 45,224 cell/mL. Significant differences between mean counts of *Microcystis* sp. in control versus treatment groups were found at days 1, 3, 7, and 14 after treatment (*P*-values <0.005). The *Microcystis* sp. counts in the treatment group decreased continually until there was only 2325 ± 1031 cell/mL, which amounted to a total overall reduction of 95.1%. While the *Microcystis* sp. counts in the control group increased by 218% over the course of the study end at 143,542 ± 66,529 cell/mL on Day 14.

Mean counts of *Scenedesmus* sp. counts for both the treatment and control groups increased over time. For the control groups, the mean count of *Scenedesmus* sp. increased from the 1-h mark to 1 day after treatment, from 16.7 ± 12.4 cell/mL to 129 ± 50.6 cell/mL, and for the time points day 1 and day 3, counts went from an average 129 ± 50.6 cell/mL to 2442 ± 857 cell/mL. The mean counts of *Scenedesmus* sp., in the treated groups decreased slightly between 1 h to 1 day post treatment, from an average of 20.8 ± 7.7 cell/mL to 12.5 ± 5.6 cell/mL and increased from day 1 to day 3 post-treatment, from 12.5 ± 5.6 cell/mL to 133 ± 69.4 cell/mL. There was no significant difference in mean counts of *Scenedesmus* sp. between treatments and controls at any time point (Fig. 2B).

**Fig. 3:**
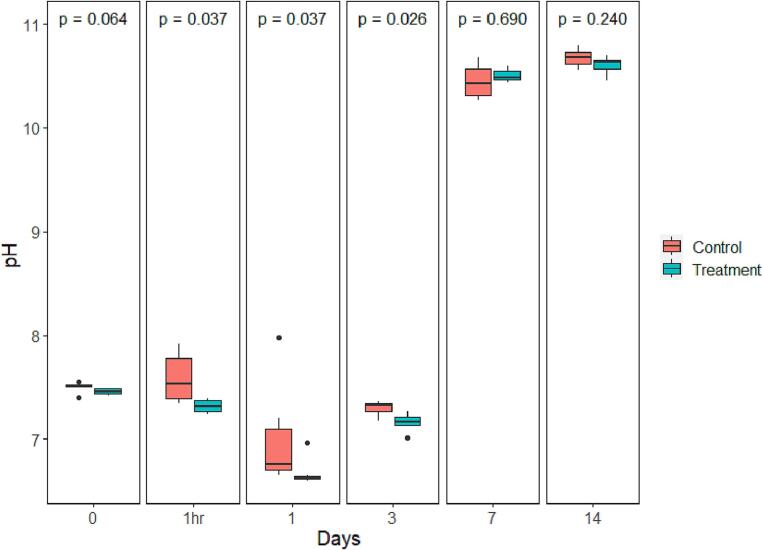
pH value comparison between the control and treatment group over time presented in box plots with median and 25–75 percentiles, the lines represent all the data range except extreme values, which are depicted with dots. *P-* value of comparison test is found at the top of the panel.

**Fig. 4:**
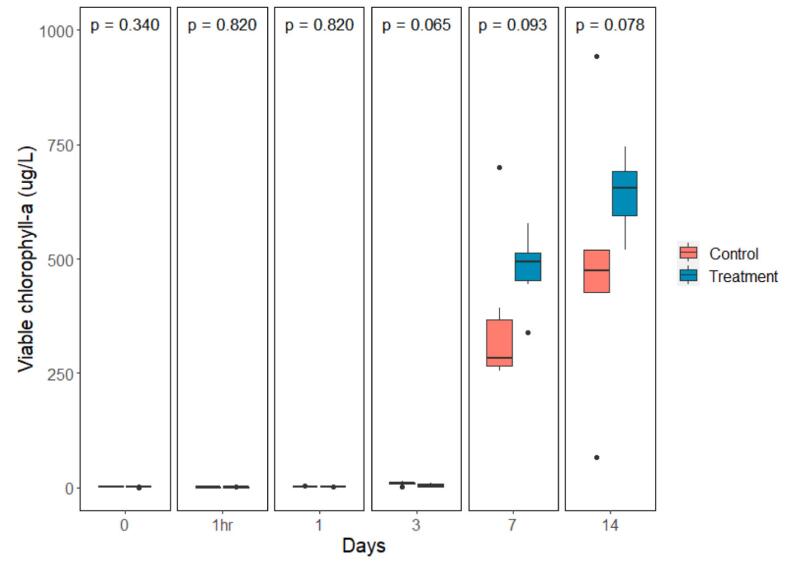
Viable chlorophyll-a concentration comparison in the control and treatment groups over time presented in box plots with median and 25–75 percentiles, the lines represent all the data range except extreme values, which are depicted with dots. *P-*value of comparison test is found at the top of the panel.

**Fig. 5:**
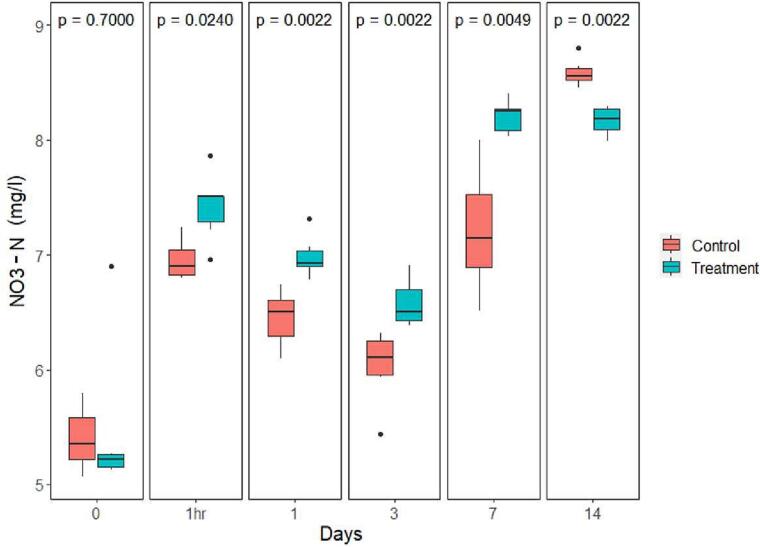
Nitrate-N concentration comparison between the control and treatment group over time presented in box plots with median and 25–75 percentiles, the lines represent all the data range except extreme values, which are depicted with dots. *P-*value of comparison test is found at the top of the panel.

**Fig. 6:**
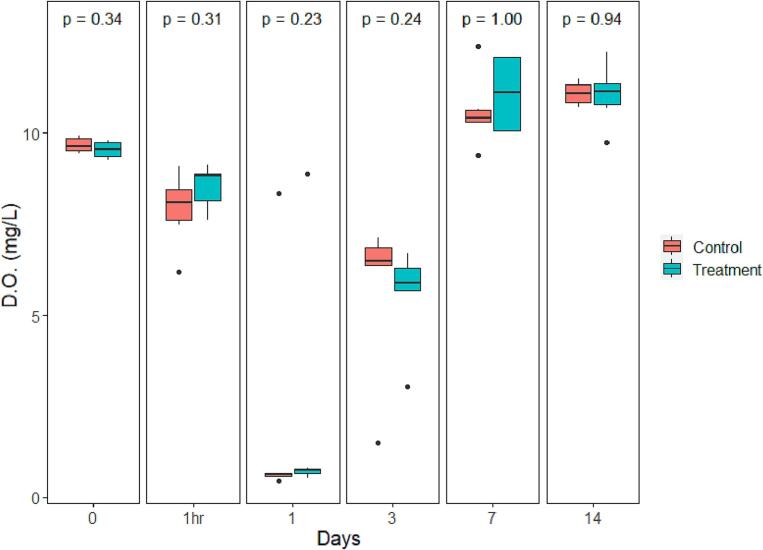
Dissolved oxygen concentration comparison between the control and treatment group over time presented in box plots with median and 25–75 percentiles, the lines represent all the data range except extreme values, which are depicted with dots. *P*-value of comparison test is found at the top of the panel.

The mean water pH levels dropped on the first day after treatment in both control and treated groups, from 7.59 ± 0.1 to 7.01 ± 0.21 and 7.32 ± 0.03 to 6.68 ± 0.06, respectively. A large increase in mean pH was visible for both groups on day 7 and day 14 after treatment, with pH levels increasing to a minimum of 10.4 for controls at day 7 and 10.6 for the treatment groups on day 14. Significant differences in mean pH occurred between the treatment and control groups at 1 h after treatment, day 1 and day 3 (*P* = 0.037, *P* = 0.037, *P* = 0.026 respectively) (Fig. 3). No statistically significant difference in mean viable chlorophyll-a was found between the treatment and control groups at any time points (Fig. 4). Much like the values observed for pH, mean viable chlorophyll-a increased sharply on day 7, stabilizing on day 14, for both control and treated samples, with the mean concentrations registered at 363 ± 70.3 μg/L for controls, and 477 ± 33 μg/L for treated samples on day 7. The mean water nitrate-N concentrations for treated and control samples increased one hour after treatment followed by a decrease on Day 1 and 3. Treated samples had a significantly higher average nitrate-N concentration compared to controls at 1 h after the treatment, and on days 1, 3 and 7 (*P* < 0.024); while the nitrate-N level in the control group was significantly higher than that of the treatment group on day 14 (*P* = 0.002) (Fig. 5). Water DO was not significantly different between control and treatment groups on any of the sampling days (Fig. 6).

### 3.2 Pond experiments

**Fig. 7:**
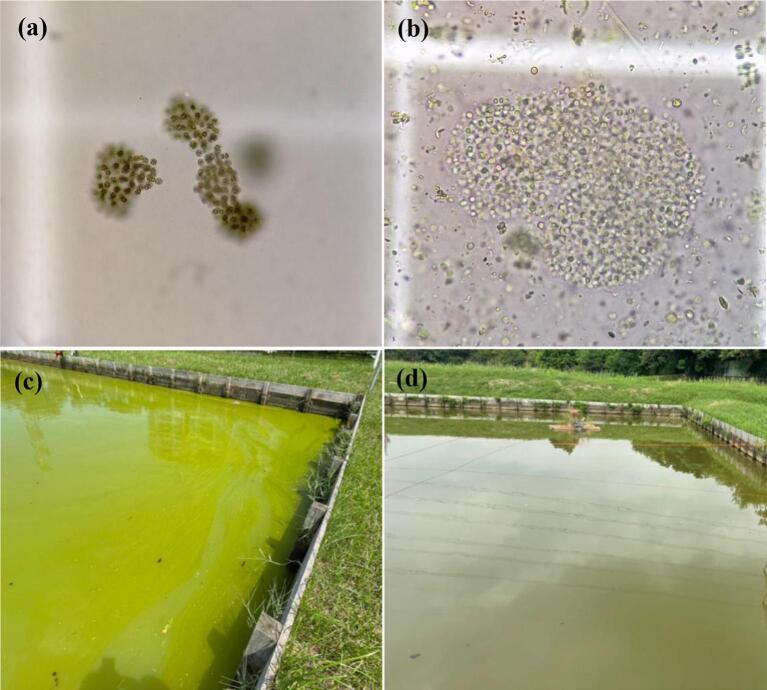
The appearance of the (a) *Microcystis* sp. colony under the light microscope before the treatment and (b) 7 days after the treatment. The appearance of the pond (c) before the treatment and (d) 7 days after the treatment.

In all pond trials the H2O2 levels one hour post treatment was approximately 7 mg/L at the water surface. Hydrogen peroxide was not detected at any other time point (Table S1). During the first trial, the initial concentration of the *Microcystis* sp. on day 0 pre-treatment was on average 251,058 ± 9075 cell/mL. There was a 23% reduction in the *Microcystis* sp. count 1 h after the H_2_O_2_ treatment (193,875 ± 14,850 cell/mL (Fig. 10d) and a second drop of 80% compared to the previous time point on 7 day after the treatment (Fig. 10d). The highest peak in the change point analysis appeared between Day 3 and Day 7 (Fig. 10a). The appearance of the treated pond changed from pear colour to olive colour after 7 days post treatment (Fig. 7c; Fig. 7d). *Microcystis* sp. colonies turned into a pale and transparent colour after treatment (Fig. 7a; Fig. 7b). However, a re-growth of the *Microcystis* sp. was detected on day 14 (2 weeks post-treatment) (Fig. 10d). The treatment did not visibly reduce the concentration of other algal species at 1 h and Day 1 compared to the pre-treatment level (Fig. 11b). The algae count excluding *Microcystis* sp. increased sharply on Day 7 (by 878%) from 19,400 ± 1229 cell/mL to 54,104 ± 3001 cell/mL, a change also detected with high probability in the change point analysis (Fig. 11d).

**Fig. 8:**
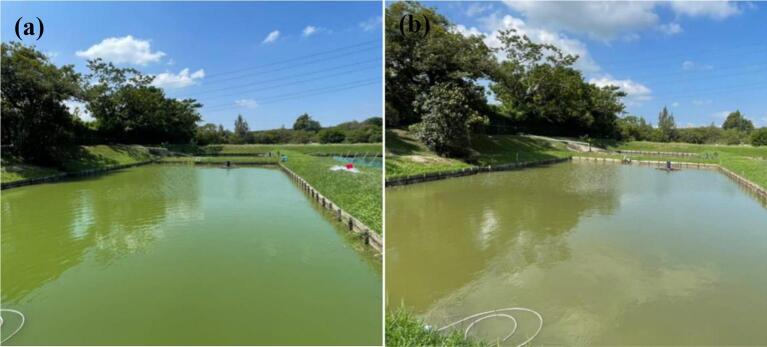
The appearance of the pond (a) before the treatment and (b) 1 day after the treatment.

**Fig. 9:**
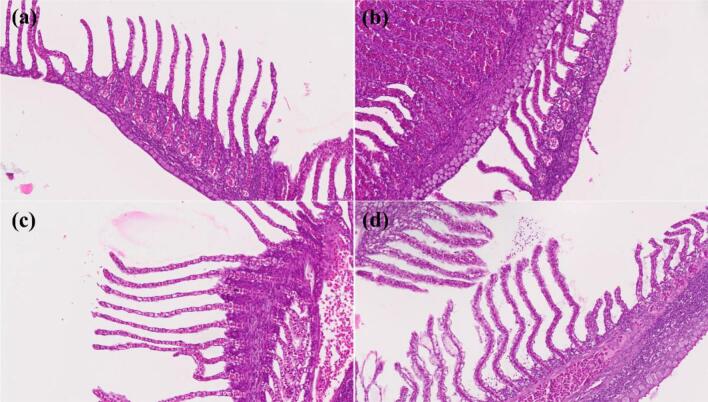
Jade perch H&E stained slides observed under the light microscope (20×). (a) Day 0, (b) 1 h post treatment, (c) Day 1, (d) Day 14.

**Fig. 10:**
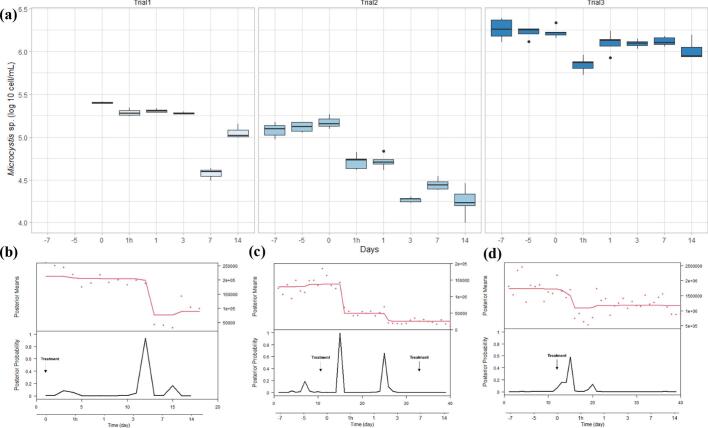
Log 10 counts of *Microcystis* sp. presented in box plots with median and 25–75 percentiles, the lines represent all the data range except extreme values, which are depicted with dots. (a) and change point analysis results, in the first (b), second (c) and third (d) pond trial, respectively.

**Fig. 11:**
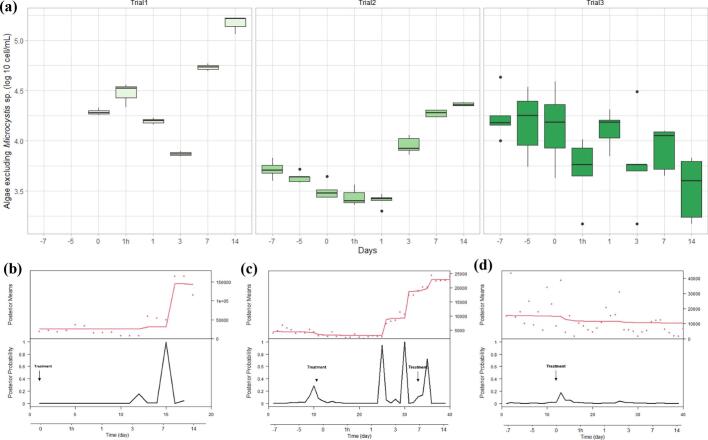
Log 10 counts of algae excluding *Microcystis* sp. presented in box plots with median and 25–75 percentiles, the lines represent all the data range except extreme values, which are depicted with dots. (a) and change point analysis results, in the first (b), second (c) and third (d) pond trial, respectively.

Both second and third pond trials did not show significant differences in *Microcystis* sp. counts on days pretreatment (i.e. Day −7, day −5, and Day 0 pretreatment). In the second trial, the *Microcystis* sp. counts dropped by 65% 1 h after the treatment (Fig. 10e) from 149,460 ± 11,417 cell/mL to 51,935 ± 3877 cell/mL, with a peak in the change point analysis indicating a very likey change in counts 1 h post treatment (Fig. 10b). A second drop of 65% occurred again on the 3rd day post treatment, with a the change point analysis indicating this drop but with a lower probability (Fig. 10b). The count of the other (non-toxic) algae after the treatment increased visibly from day 1 to day 3 after treatment, also signalled by the change point analysis with high posterior probability (Fig. 11a; Fig. 11d). The colour of the pond changed from a jade to olive colour one day after the treatment (Fig. 8). During the twenty-one days investigation period, one fish among the four-hundred Jade perch in the treated pond died on the seventh day. No H_2_O_2_ treatment-related health issues were grossly observed on this individual (Fig. 9).

**Fig. 12:**
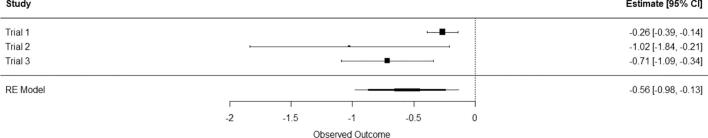
Time series GLM summary statistics performed on *Microcystis* sp., counts with intervention effect at 1 h, day 1 and day 3, and a lag of 1. A forest plot of the coefficients for the GLMs pooling three trial coefficient estimates for the treatment effect provides a global estimate of the reduction in *Microcystis* sp. counts over the 3 day period after treated with hydrogen peroxide. A negative observed outcome represents the reduction of *Microcystis* sp., and [1- e^(CI)] x100% shows the degree of reduction.

**Fig. 13:**
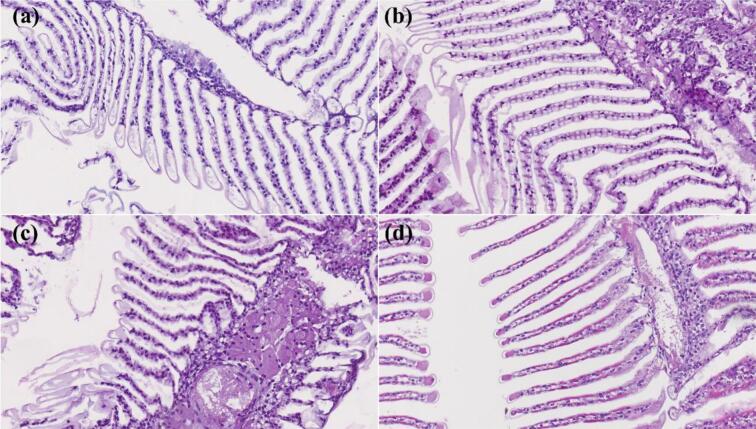
Giant tiger prawn H&E stained slides observed under the light microscope (20×). (a) Day 0, (b) 1 h, (c) Day 1, (d) Day 14.

In the third trial, the *Microcystis* sp. counts dropped by 58% 1 h after the treatment from 1,701,190 ± 158,902 cell/mL to 713,086 ± 45,978 cell/mL (Fig. 10f). However, a regrowth of *Microcystis* sp. was found on Day 1 with a count of 1,293,356 ± 118,121 cell/mL (Fig. 10f). The *Microcystis* sp. counts were at 1,060,410 ± 131,645 cell/mL on Day 14. The highest peak in posterior probability of our change point analysis occurred between Day 0 and 1 h (Fig. 10c). GLM fits for the three trials produced negative coefficients for the intervention variable, indicating a significant reduction in *Microcystis* sp. counts over the three day period of analysis. Metanalysis of the time-series GLM fits for the three pond trials also suggest a weighted average overall reduction of *Microcystis* sp. of 43% associated with hydrogen peroxide treatments during the three day time frame(Fig. 12). No significant damage on the shrimp gills were found during the experimental period in this pond (Fig. 13).

**Fig. 14:**
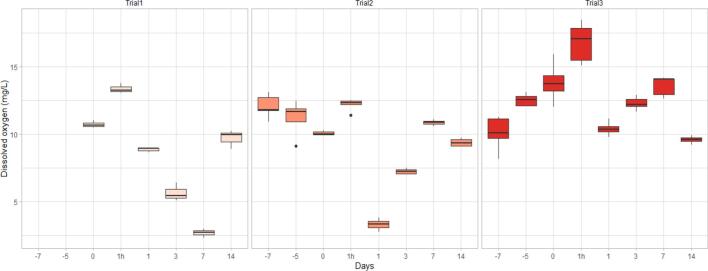
DO changes over time in Trial 1, Trial 2 and Trial 3 presented in box plots with median and 25–75 percentiles, the lines represent all the data range except extreme values, which are depicted with dots.

### 3.3 Water quality

In the three pond trials, DO was higher 1 h after the treatment compared to pretreatment levels (Trial 1: 10.72 ± 0.15 mg/L to 13.38 ± 0.16 mg/L; Trial 2: 10.07 ± 0.05 mg/L to 12.18 ± 0.14 mg/L; Trial 3: 13.85 ± 0.47 mg/L to 16.81 ± 0.49 mg/L) and dropped visibly on Day 1 post treatment (Trial 1: 10.72 ± 0.15 mg/L to 8.86 ± 0.08 mg/L; Trial 2: 10.07 ± 0.05 mg/L to 3.31 ± 0.18 mg/L; Trial 3: 13.85 ± 0.47 mg/L to 10.42 ± 0.16 mg/L) (Fig. 14).

The pH of the water in the first and second pond trials dropped 1 day after the treatment (Trial 1: 9.64 ± 0.01 mg/L to 9.38 ± 0.01 mg/L; Trial 2: 9.35 ± 0.03 mg/L to 7.69 ± 0.02 mg/L). However, only a relatively minor drop of pH was found in trial 3 on Day 1(8.74 ± 0.08 mg/L to 8.42 ± 0.11 mg/L) (Fig. 15).

## 4 Discussion

Application of H_2_O_2_ to earthen ponds is a promising method of reducing *Microcystis* sp. as demonstrated in this study. We observed an overall average 43% reduction in *Microcystis* sp. in our pond trials over the first three days post H_2_O_2_ treatment. The immediate treatment effect after 1 h was much greater with one pond (trial 2) having a 65% reduction in *Microcystis* sp.; however, there was some variation in the initial drop across trials. At the same time, other beneficial algal species increased after treatment, so H_2_O_2_ at the concentration of 7 mg/L appeared to be effective specifically against *Microcystis* sp., but had no effect on other types of algae. This was also observed in our laboratory experiments. Further, the amount of H_2_O_2_ to reduce cyanobacteria did not affect fish and crustaceans in our two test ponds.

The mechanism of action of H_2_O_2_ on cyanobacteria has yet to be fully understood. Piel et al. (2019) suggest H_2_O_2_ disrupts and impairs the repair of the D1 protein located in the lumen of the thylakoid, which are not protected by chloroplasts naturally found in other types of algae. This rapid turnover protein is one of the key component of photosystem II as it is the primary electron acceptor to convert light energy to chemical energy (Inagaki, 2022; Mamedov et al., 2015). Hydrogen peroxide may also prevent algae from repairing or replacing damaged D1 proteins. It is believed that the precursor D1 protein (pD1), which would replace damaged D1 protein is activated by the assemblage of the CP43 protein and the water-oxidizing complex Mn_4_CaO_5_ (Inagaki, 2022; Theis and Schroda, 2016). Pham and Messinger (2014) reported that H_2_O_2_ acts as an oxidant and reductant to Mn_4_CaO_5_, which may result in the disruption of the replacement of D1 proteins, and lead to issues with photosynthesis. Spoof et al. (2020) also reported that H_2_O_2_ had a negative impact on electron transfer and oxygen production associated with photosynthesis in cyanobacteria. The hypothesized mechanisms for the destruction of cyanobacteria may explain the selective effect of H_2_O_2_ on this group of algae. Other algal species with chloroplast may be more resistance to the oxidative stress associated with the concentration of H_2_O_2_ used in our experiments (Chai et al., 2005). We also confirmed the lack of effect on *Scenedesmus* sp.in our laboratory study. Further research could be done to elucidate the concentrations necessary for killing other algae species. In addition further studies regarding the effectiveness of H_2_O_2_ when varying levels of chlorophyll, total suspended solids, and volatile suspended solids are in the water would be helpful for adjusting concentrations of H_2_O_2_ for treatment purposes. Buley et al. (2023) suggested that the initial algal biomass could impact the effectiveness of H_2_O_2._

Other studies have demonstrated another advantage of using H_2_O_2_ for controlling cyanobacteria in ponds is that it enhances the photocatalytic destruction of the microcystin toxins released when these algae are damaged (Cornish et al., 2000; Wang et al., 2015). Neutralization of the toxins produced by the cyanobacteria is important in aquaculture ponds with live animals. Our findings are consistent with the fact that killing cyanobacteria in ponds did not impact fish or crustaceans in a negative way in our study. However, our findings were limited to two ponds so they should be interpreted with caution.

One of the reasons for farmers to treat ponds with cyanobacteria, besides the fact that this algae can be toxic to fish, is that high levels of algae in ponds can cause fluctuations in DO and pH, which in extreme circumstances could result in health issue (Jensen et al., 2011; Kemka et al., 2003; Lopez-Archilla et al., 2004). The fluctuations in water chemistry are due to the change between aerobic and anaerobic respiration of algae during the day and night cycles, respectively (i.e. High CO_2_ and low DO at night; High DO and low CO_2_ in the day) (Cuaresma et al., 2011; Edmundson and Huesemann, 2015). In our pond studies, we found that the daytime DO levels increased slightly 1 h post treatment, but decreased within one day. The initial increase may have been due to the breakdown of H_2_O_2_ into oxygen and water, as well as the natural O_2_ production from photosynthesis of the algae in the pond, as it may take more than one hour to have the full treatment effect. The decline in daytime DO and pH levels over the few days following the treatment likely reflected the reduction in algae cells in the pond.

One potential issue with using H_2_O_2_ may be a change in pH. In our study, we measured a decrease in pH during the treatment, especially in trial 2. To circumvent this drop which was assumed to be related by the addition of H_2_O_2_, the treatment could be applied with a buffer (Bergstedt et al., 2022; Powell et al., 2015). This may be especially important if for ponds with low carbonate hardness (KH). In our ponds, KH was above 90 mg/L during the treatment so we did not administer a buffer with our low dose H_2_O_2_ treatment, but the fact that we observed a slight decrease in pH suggests that it might be better in the future to add a buffer such as sodium bicarbonate.

A regrowth of algae was found in our treated ponds on day 7. Fortunately, it was mostly associated with an increase in non-toxic algae. However, we observed a regrowth of *Microcystis* sp. on day 14 in trial 1. In trial 2, we tried to mitigate this by administrating a second H_2_O_2_ treatment on day 7. Although no controls were present in this situation, we did not observe regrowth of *Microcystis* sp. after the second treatment. The use of a second H_2_O_2_ treatment may be useful to inhibit the regrowth of *Microcystis* sp. if cell counts start increasing post treatment. Future research may also investigate the use of higher concentrations of H_2_O_2_ to increase the duration of effectiveness of the treatment. However, if the regrowth of algae is a result of non-cyanobacteria algae, then as our results suggests 7 mg/L of H_2_O_2_ will not work against these forms of algae, and farmers will have to resort to other types of treatments that may not be as environmentally friendly as H_2_O_2_ but which have a broader treatment spectrum.

The dose of H_2_O_2_ used in this study was low and safe for both the animals in the pond and the users; However, the initial product purchased was highly concentrated (i.e. 30% H_2_O_2_) in order to achieve the desired concentration in the pond. At this concentration, H_2_O_2_ is very caustic and can be dangerous for farmers to handle. Further, the larger the pond the more difficult it is to distribute the chemical evenly to achieve the required dose for the effect. In our ponds, which were small, we were able to design a distribution device to facilitate the application of this chemical. While in larger ponds, a boat or the use of other application methods may be required.

Overall, the findings from our pond study suggest that H_2_O_2_ could be very useful for farmers to control cyanobacteria; however, the lack of a proper control group limits our conclusions. The difficulty in conducting clinical control trials in a natural setting is that every pond is unique regarding cyanobacteria counts, buffering capability and organic loading so it is difficult to find controls and replicates. To overcome this limitation, we conducted the study in three ponds, and we analysed the change in *Microcystis* sp. over time using time 0 as a control. An intervention effect was estimated using a GLM model and the results of the three trials were aggregated for the weighted overall effect. In addition, we refer to the results of the laboratory experiment to confirm the effect of H_2_O_2_ on specific species of algae. Taking into consideration the results of all experiments reported in this study, the findings strongly indicate that low doses of H_2_O_2_ is effective at reducing *Microcystis* sp. and has limited impact on other algae and aquatic life.

## 5 Conclusions

The concentration of H_2_O_2_ (7 mg/L) used in this study was effective at reducing *Microcystis* sp. in earthen ponds within 1 h, and this was corroborated by our laboratory studies. The treatment did not affect the water quality of earthen ponds other than pH, which could be mitigated with a buffer; however, the reduction in the cyanobacteria might lead to lower DO during the daytime due to reduced photosynthetic activity. No cellular damage was observed in the gills of tilapia or giant tiger prawn suggesting the application of H_2_O_2_ at the concentration used in this study may be a good option to reduce toxic cyanobacteria from aquaculture ponds. However, treatments may need to be repeated weekly or biweekly depending on the particular pond ecosystem and success of the initial treatments.

## Funding

This work was supported by the Agriculture, Fisheries and Conservation Department (AFCD), Hong Kong SAR, China [grant number: SFDF_0042] and the 10.13039/100013986UK government – 10.13039/501100003921Department of Health and Social Care (DHSC), 10.13039/100012774Global AMR Innovation fund (GAMRIF) and the International Development Research Center (IDRC), Ottawa, Canada.

## CRediT authorship contribution statement

**Pok Him Ng:** Conceptualization, Formal analysis, Methodology, Writing – original draft. **Tzu Hsuan Cheng:** Investigation. **Ka Yan Man:** Investigation. **Liqing Huang:** Investigation. **Ka Po Cheng:** Investigation. **Kwok Zu Lim:** Investigation. **Chi Ho Chan:** Investigation. **Maximilian Ho Yat Kam:** Investigation. **Ju Zhang:** Investigation. **Ana Rita Pinheiro Marques:** Formal analysis, Writing – review & editing. **Sophie St-Hilaire:** Supervision, Writing – original draft.

## Declaration of Competing Interest

The authors declare the following financial interests/personal relationships which may be considered as potential competing interests:

POK HIM NG reports financial support and equipment, drugs, or supplies were provided by Agriculture, Fisheries and Conservation Department (AFCD), Hong Kong SAR, China.

## Data Availability

Data will be made available on request.

## Appendix A Supplementary data

Supplementary material Image 1
