# Corrigendum to “Hydrogen peroxide as a mitigation against *Microcystis* sp. Bloom” [Aquaculture, Volume 577, 15 December 2023, 739932]

**DOI:** 10.1016/j.aquaculture.2024.741655

**Published:** 2025-01-30

**Authors:** Pok Him Ng, Tzu Hsuan Cheng, Ka Yan Man, Liqing Huang, Ka Po Cheng, Kwok Zu Lim, Chi Ho Chan, Maximilian Ho Yat Kam, Ju Zhang, Ana Rita Pinheiro Marques, Sophie St-Hilaire

**Affiliations:** Department of Infectious Diseases and Public Health, Jockey Club College of Veterinary Medicine and Life Sciences, City University of Hong Kong, Hong Kong, China

**Figure:**
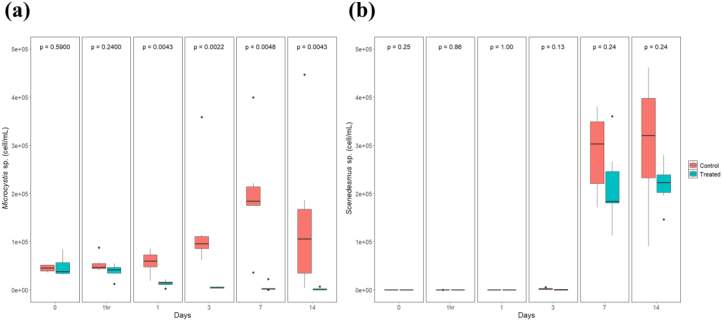

The authors regret in Fig. 2 (a) from the “Results” section, the color of the Treated and Control group is reversed. Below is the revised Fig. 2.

The authors would like to apologise for any inconvenience caused.

