# Supplementary material for: Hydrogen peroxide as a mitigation against Microcystis sp. bloom
Source: Aquaculture. 2023 Dec 15;577:739932. doi: 10.1016/j.aquaculture.2023.739932 (PMC10518459; doi:10.1016/j.aquaculture.2023.739932)
Supplement: Supplementary file 1 — Supplementary material [file mmc1.docx]

# **Supplementary materials**

*Laboratory study*


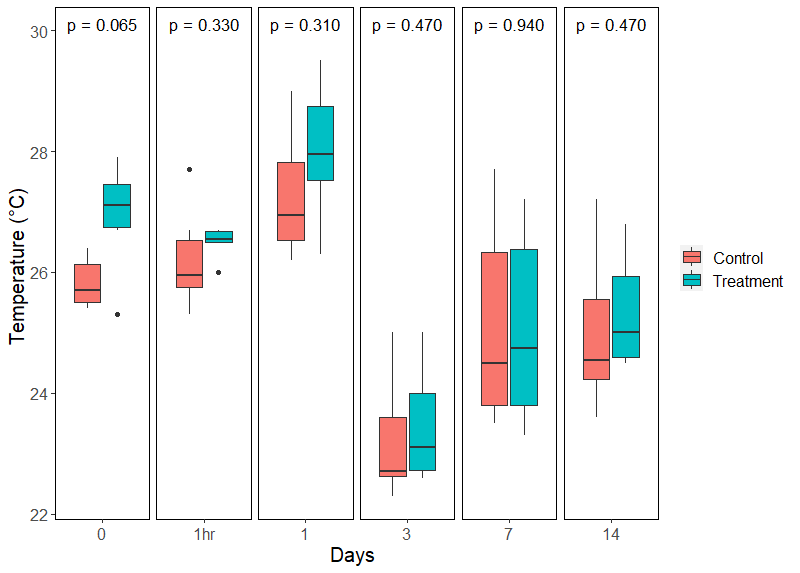


Fig S1. Temperature comparison between the control and treatment group over time presented in box plots with median and 25 – 75 percentiles, the lines represent all the data range except extreme values, which are depicted with dots.

*Pond study*


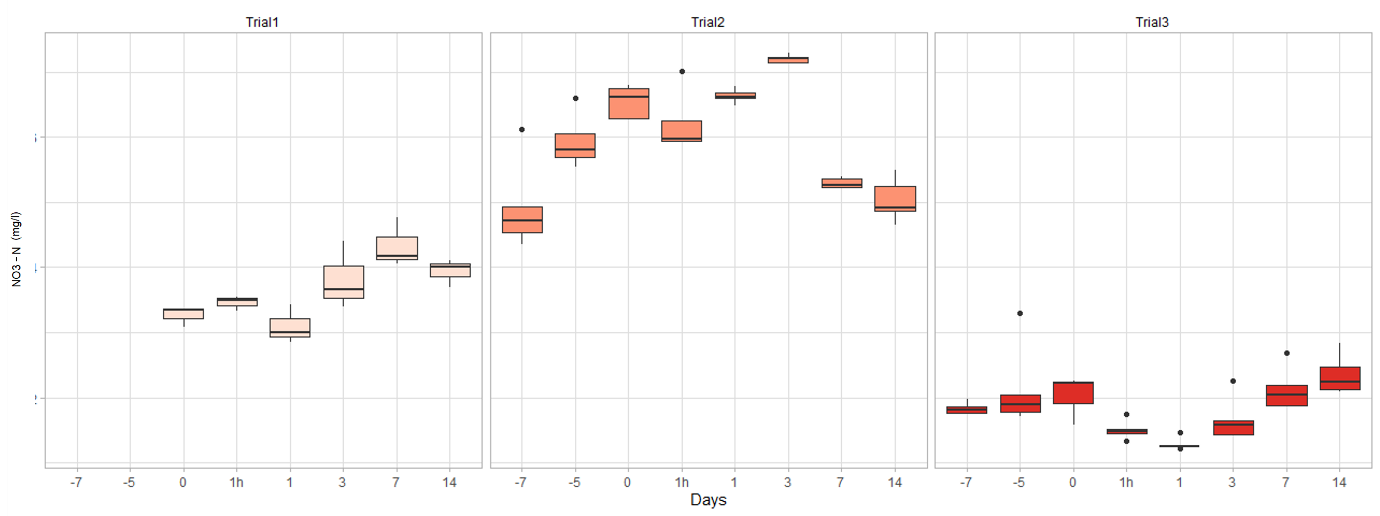


Fig S3. Nitrate-N changes over time in Trial 1, Trial 2 and Trial 3 presented in box plots with median and 25 – 75 percentiles, the lines represent all the data range except extreme values, which are depicted with dots.


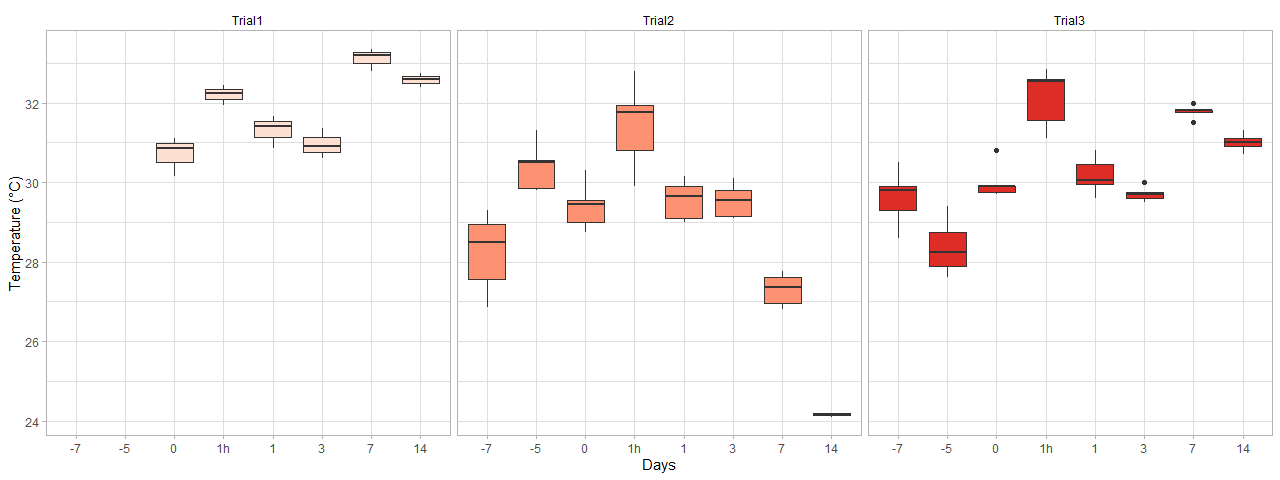


Fig S2. Temperature changes over time in Trial 1, Trial 2 and Trial 3 presented in box plots with median and 25 – 75 percentiles, the lines represent all the data range except extreme values, which are depicted with dots.


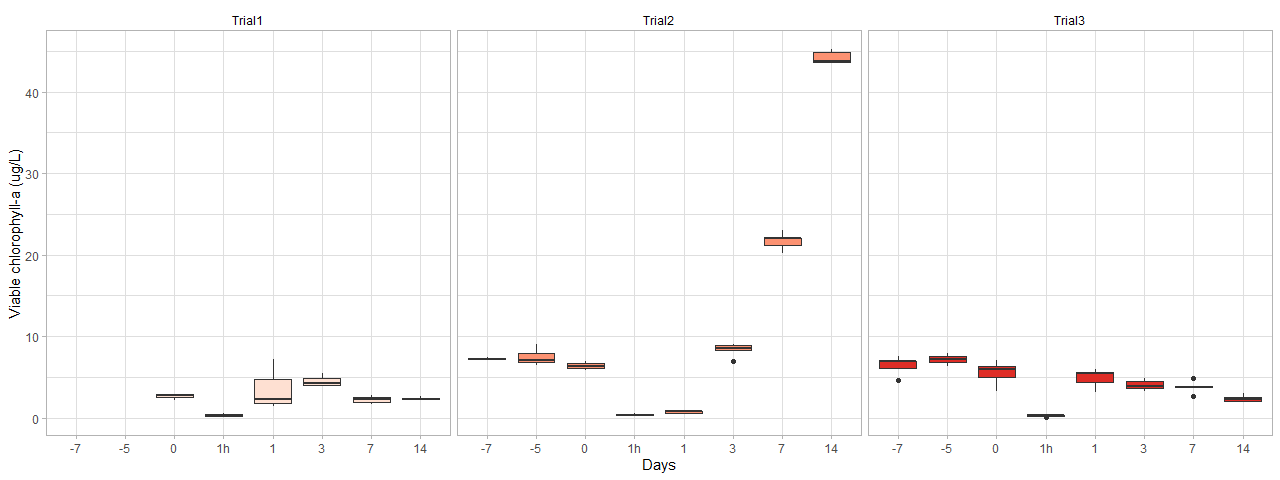


Fig S4. Viable chlorophyll-a changes over time in Trial 1, Trial 2 and Trial 3 presented in box plots with median and 25 – 75 percentiles, the lines represent all the data range except extreme values, which are depicted with dots.

Table S1. Average hydrogen peroxide concentration with standard error in Trial 1, Trial 2 and Trial 3.

| **Study** |  | **Hydrogen peroxide mean concentration**  **(mg/L)** | **Std. err.** |  |
| --- | --- | --- | --- | --- |
|  |  |  |  |  |
|  |  |  |  |  |
| **Pond trial 1** |  |  |  |  |
|  | Day 0 | 0.000 | 0.000 |  |
|  | 1hr after the treatment | 7.250 | 0.582 |  |
|  | 1 day after the treatment | 0.000 | 0.000 |  |
|  | 3 days after the treatment | 0.000 | 0.000 |  |
|  |  |  |  |  |
| **Pond trial 2** |  |  |  |  |
|  | Day 0 | 0.000 | 0.000 |  |
|  | 1hr after the treatment | 7.000 | 0.000 |  |
|  | 1 day after the treatment | 0.000 | 0.000 |  |
|  | 3 days after the treatment | 0.000 | 0.000 |  |
|  |  |  |  |  |
| **Pond trial 3** |  |  |  |  |
|  | Day 0 | 0.000 | 0.000 |  |
|  | 1hr after the treatment | 7.000 | 0.211 |  |
|  | 1 day after the treatment | 0.000 | 0.000 |  |
|  | 3 days after the treatment | 0.000 | 0.000 |  |
